# Kynurenine promotes the immune escape of colorectal cancer cells via NAT10-mediated ac^4^C acetylation of PD-L1

**DOI:** 10.1016/j.clinsp.2025.100658

**Published:** 2025-04-16

**Authors:** Zaibiao Wang, Manman Yin, Ruhang Zhou, Ming Li, Jie Peng, Zhengguang Wang

**Affiliations:** a Department of General Surgery, The First Affiliated Hospital of Anhui Medical University, Hefei, Anhui, PR China; b Department of General Surgery, The Affiliated Bozhou Hospital of Anhui Medical University, Bozhou, Anhui, PR China; c Department of Science and Education, The Affiliated Bozhou Hospital of Anhui Medical University, Bozhou, Anhui, PR China; d Department of General Surgery, The Second Affiliated Hospital of Anhui Medical University, Hefei, Anhui, PR China; e Department of Pathology, The Affiliated Bozhou Hospital of Anhui Medical University, Bozhou, Anhui, PR China

**Keywords:** Kynurenine, Colorectal cancer, N4-acetylcytidine modification, N-acetyltransferase 10, Immune escape, Programmed death-ligand 1

## Abstract

**Background:**

This study aimed to investigate the role of kynurenine in Colorectal Cancer (CRC) and the underlying mechanism.

**Methods:**

Enzyme-linked immunosorbent assay was employed to assess the kynurenine concentration. Flow cytometry was utilized to analyze the percentages of CD3+CD4+ and CD3+CD8+ *T*-cells. Immunofluorescence was used to measure the expression of Programmed Death-Ligand 1 (PD-L1). RNA modification levels in CRC cells were analyzed using a dot blot assay. The interaction between NAT10 and PD-L1 was assessed via RNA immunoprecipitation, dual-luciferase reporter, and immunofluorescence assays. A xenograft tumor rat model was established.

**Results:**

Results indicated that kynurenine suppressed T-cell activation and promoted immune escape. Besides, kynurenine promoted N-Acetyltransferase 10 (NAT10)-mediated N4-acetylcytidine (ac^4^C) modification. Moreover, NAT10 inhibition improved T-cell activation and suppressed immune escape. Mechanically, NAT10 is bound with the mRNA of PD-L1. Rescue experiments showed that PD-L1 inhibitor treatment reversed the suppressed T-cell activation and the promoted immune escape induced by NAT10 overexpression. In vivo, studies indicated that NAT10 deficiency reversed the promoted tumor growth induced by kynurenine treatment.

**Conclusion:**

In conclusion, kynurenine promoted the immune escape of CRC cells via NAT10-mediated ac^4^C acetylation of PD-L1.

## Highlights

• Kynurenine suppressed T-cell activation and promoted immune escape.
• Kynurenine promoted NAT10-mediated ac^4^C mRNA modification.
• NAT10 inhibition improved T-cell activation and suppressed immune escape.

## Introduction

Colorectal Cancer (CRC), a common malignancy of the gastrointestinal tract, develops from abnormal growth of polyps formed in the inner lining of the colon or rectum.^1^ Over time, these polyps undergo malignant transformation, invade nearby tissues or spread to other parts of the body, thus leading to the progression of CRC.^2^ The precise etiology of CRC remains incompletely elucidated; however, numerous risk factors have been recognized, encompassing increasing age, hereditary susceptibility, a personal medical history of inflammatory bowel disease, obesity, smoking, and physical inactivity.^2^ The symptoms of CRC vary depending on the location and stage of the cancer. Common symptoms include changes in diarrhea, constipation, blood in the stool, abdominal pain or cramping, fatigue, and a feeling of incomplete bowel movements.^3^ Therapeutic approaches for CRC include surgery, chemotherapy, radiation therapy, targeted therapy, or immunotherapy, but the mortality and recurrence rates are still high.^1^ Thus, understanding the pathogenesis of CRC is critical to improving outcomes and reducing the burden of CRC.

Kynurenine plays a crucial role as a metabolite within the kynurenine pathway, where it facilitates the degradation of tryptophan, an indispensable amino acid. The conversion of tryptophan into kynurenine is catalyzed by either Tryptophan 2,3-Dioxygenase (TDO) or Indoleamine 2,3-Dioxygenase (IDO), depending on the specific tissue and cellular environment.^4^ Kynurenine is involved in the progression of various cancer types due to its ability to regulate the immune response and promote tumor growth.^5^ It has been shown to inhibit the activity of a variety of immune cells, such as T-cells and natural killer cells.^5^ In addition, kynurenine can induce differentiation of regulatory T-cells, thereby further inhibiting the immune response of tumors.^6^ Thus, the accumulation of kynurenine in the tumor microenvironment creates an immunosuppressive and pro-tumor niche that supports cancer progression. Targeting the kynurenine pathway or its downstream effects might provide novel therapeutic strategies for anticancers.

An increasing number of RNA modifications, including N4-acetylcytidine (ac^4^C), 5-methylcytosine (m^5^C), N6-methyladenosine (m^6^A), and 7-methylguanosine (m^7^G), have been widely discovered in cancers. ac^4^C modification forms by adding an acetyl group to cytidine nucleotides. This modification plays a crucial role in regulating various aspects of mRNA function and stability, including mRNA translation, splicing, and degradation.^7^ N-Acetyltransferase-like protein-10 (NAT10) is the first enzyme discovered in eukaryotic RNA to catalyze the generation of ac^4^C, possessing acetyltransferase activity and RNA binding activity.^7^ NAT10-mediated ac^4^C modification promotes the progression of different cancers, including CRC.^7^^,^^8^

Tumor immune evasion refers to the phenomenon in which tumor cells evade recognition and attack by the immune system through various mechanisms, allowing them to grow and metastasize.^9^ It is an important strategy for the survival and development of tumors. Tumor-induced immune suppression is currently the most extensively studied immune evasion mechanism. Additionally, Programmed Cell Death Ligand-1 (PD-L1), a transmembrane protein that binds to programmed cell death Protein-1 (PD-1), reduces the proliferation of PD-1-positive cells and induces cell apoptosis.^9^ On the other hand, PD-L1 causes dysfunction in T-cell function and binds to PD-1 on T-cells, preventing cytotoxic T-cells from effectively targeting tumor cells, thereby promoting tumor occurrence and development. Increasing evidence suggests that the PD-1/PD-L1 axis is responsible for immune evasion in cancer and accelerates the growth of multiple tumors.^10^ However, the relationship between NAT10 and immune evasion in CRC is largely unknown.

In this study, the authors aimed to investigate the expression and effects of kynurenine in CRC and its underlying mechanism, which might provide a theoretical basis for the treatment of CRC.

## Methods and materials

### *Cell culture*

Human normal colonic epithelial cells (Cat. #FH0541; NCM460) and CRC cell lines (HCT116 [Cat. #FH0027], DLD-1 [Cat. #FH0017], SW480 [Cat. #FH0022], SW620 [Cat. #FH0021], and RKO [Cat. #FH0030]) were obtained from Fuheng Biotechnology Co., LTD (Shanghai, China) and cultured in their specified complete mediums (Fuheng). All cells were cultured in an incubator with 37 °C and 5 % CO2.

### *Cell transfection and treatment*

Short hairpin (sh) negative control (shNC) plasmid, shNAT10 plasmid, negative control pcDNA 3.1 vectors, and pcDNA 3.1-NAT10 overexpression vector were synthesized by Ribio Biotechnology Co., LTD (Guangzhou, China). DLD-1 and SW480 cells were seeded at a density of 4 × 10^5^ cells per well in 6-well plates. Once the cell confluence reached 80 %, transfection was carried out for 48 h using the PolyFast transfection reagent (Cat. #HY-K1014; MedChemExpress, Monmouth Junction, NJ, USA). DLD-1 and SW480 cells were treated with kynurenine (Cat. #K8625; 50 μM; MedChemExpress) for 4 h or αPD-L1 (Cat. #SIM0009; 15 µg/mL; BioXCell, West Lebanon, NH, USA), a PD-L1 inhibitor, for 48 h.

### Reverse transcription-quantitative polymerase chain reaction (RT-qPCR)

Total RNA was isolated from cells using the commercial MolPure® Cell RNA kit (Cat. #19221ES; Yeason Biotechnology Co., LTD, Shanghai, China). The commercial Hifair® Ⅲ Reverse Transcriptase kit (Cat. #14601ES; Yeason) was utilized for the reverse transcription process in cDNA synthesis. The amplification of qPCR was performed utilizing the commercial Hieff® qPCR SYBR Green Master Mix kit (Cat. #11201ES; Yeason) following the prescribed reaction conditions. The primers used in this study were obtained from Thermo Fisher Scientific (Waltham, MA, USA), and are listed below: N-Acetyltransferase (NAT) 10, forward, 5′-ATAGCAGCCACAAACATTCGC-3′ and reverse, 5′-ACACACATGCCGAAGGTATTG-3′; Programmed Death-Ligand 1 (PD-L1), forward, 5′-GCCAGAAAAGCCTCATTCGT-3′ and reverse, 5′-TGAATCTCGAAACCTCCAGGAA-3′; Glyceraldehyde-3-Phosphate Dehydrogenase (GAPDH), 5′-TGTGGGCATCAATGGATTTGG-3′ and reverse, 5′-ACACCATGTATTCCGGGTCAAT-3′. The gene expression was calculated by the 2^−ΔΔCT^ method and GAPDH was used as the internal control.

### *Measurement of kynurenine concentration*

Kynurenine from CRC cells was quantified using the commercial kynurenine enzyme-linked immunosorbent assay (ELISA) kit (Cat. #ELK9026; ELK Biotechnology Co., LTD, Wuhan, China). All operations were carried out in strict accordance with the instructions.

### *MTT assay*

The cytotoxicity of kynurenine on DLD-1 and SW480 cells was evaluated by a commercial MTT kit (Cat. #C0009S; Beyotime Biotechnology Co., LTD, Shanghai, China). Firstly, DLD-1 and SW480 cells were seeded (5 × 10^3^ cells/well) in a flat-bottom 96-well plate and incubated in an incubator at 37 °C, and 5 % CO_2_. Subsequently, 10 μL of the MTT solution was introduced into each well and incubated in the above incubator for 4 h. Following this, 100 μL of Formazan solution was added to each well, thoroughly mixed, and incubated in the incubator for another 4 h until the Formazan was fully dissolved. Finally, the absorbance at 570 nm was assessed utilizing a microplate reader (Thermo Fisher).

### *Flow cytometry*

Flow cytometry was performed according to a previous study.^11^ Firstly, The DLD-1 and SW480 cells (5 × 10^5^) were washed twice with cell staining buffer (Cat. #420,201; Biolegend, San Diego, CA, USA) and were subsequently fixed using fixation buffer (Cat. #40402ES50; Yeason). The cell membranes were broken using a permeabilization buffer (Cat. #40403ES64; Yeason). Next, DLD-1 and SW480 cells single-cell suspensions were incubated with Fluorescein 5-Isothiocyanate (FITC)-conjugated anti-human CD3 (Cat. #16–0037–81; Thermo Fisher), Allophycocyanin (APC)-conjugated anti-human CD4 (Cat. #11–0049–42; Thermo Fisher) and Phycoerythrin (PE)-conjugated anti-human CD8 (Cat. #56–0088–42; Thermo Fisher) antibodies at 4 °C for 30 min. The suspensions were then washed with sheath fluid. Flow cytometry was performed using CytoFLEX LX (Beckman, Brea, CA, USA) and analyzed using FlowJo v10 software.

### *Immunofluorescence (IF) staining*

The cells cultivated on a glass slide were immersed in Phosphate Buffer Saline (PBS, pH 7.4; Cat. #10,010,023; Thermo Fisher) thrice and then fixed with 4 % paraformaldehyde (Cat. #P0099; Beyotime) for 15 min. Subsequently, the sections were washed with PBS thrice and incubated with the PD-L1 (Cat. #PA5–20,343; Thermo Fisher) and NAT10 (Cat. #ab194297; Abcam, Cambridge, MA, USA) antibodies overnight at 4 °C. This was followed by incubation with the secondary antibody (Cat. #A32740; Thermo Fisher) for 1 h without light at room temperature. Then, the sections were mounted with an Antifade Mounting solution containing 10 mg/mL 4′6-diamidino-2-phenylindole (DAPI; Cat. #C1005; Beyotime). Representative visual fields were acquired via the DM5000 B microscope (Leica Microsystems, Wetzlar, Germany).

### *Western blot*

Total proteins were extracted using the commercial RIPA buffer (Cat. #P0013B, Beyotime) and quantified via a BCA protein assay kit (Cat. #20201ES76, Yeason). Next, 30  μg of proteins were separated by 10 % SDS-PAGE and transferred to a polyvinylidene fluoride membrane. After blocked with Fast Blocking Western (Cat. #36122ES60; Yeason) for 10 min and incubated with the primary antibodies (NAT10 [Cat. #ab194297; Abcam], PD-L1 [Cat. #ab205921; Abcam], and GAPDH [Cat. #ab9485; Abcam]) at 4 °C overnight, followed by HRP-conjugated goat-anti-rabbit secondary antibody (Cat. #ab6721; Abcam) incubation. Finally, an enhanced chemiluminescence solution (Cat. #32,106; Thermo Fisher) was used for protein signal detection.

### *Dot blot assay*

Initially, RNA extracted from DLD-1 and SW480 cell samples underwent a heat treatment at 95 °C for 3 min. The concentration of RNA was determined using a Nanodrop 2000 (Thermo Fisher). Subsequently, total RNA was separately applied to a nitrocellulose filter membrane (Abcolne, CN) and dried at 37 °C for 30 min. Following this, the membrane was washed with Tris Buffered Saline Tween (TBST) for 5 min to eliminate any unbound RNA. After removing the TBST, the sample was blocked with 5 % bovine serum albumin for 1 h, and then incubated with ac^4^C (Cat. #ab252215; Abcam), m^5^C (Cat. #68,301–1-Ig; Proteintech Biotechnology Co. LTD, Wuhan, China), m^6^A (Cat. #ab314476; Abcam), and m^7^G (Cat. #ab300740; Abcam) antibodies overnight at 4 °C. The membrane was then washed thrice with TBST for 10 min each. Finally, the samples were treated with a secondary antibody (Cat. #ab6721; Abcam) for 1 h, and the signals from the dot blot were visualized using an ECL reagent (Cat. #ab65623; Abcam) for 1 min.

### *Ac^4^C-RNA immunoprecipitation (RIP)-qPCR*

Firstly, total RNA was isolated from DLD-1 and SW480 cell samples. The ac^4^C-RIP assay was performed using the GenSeq ac^4^C RIP kit (Cat. #GS-ET-005, Cloudseq Biotech, Shanghai, China) in accordance with the published literature.^12^ RNA samples were fragmented into 100-nucleotide-long using RNA Fragmentation reagents (Cat. #AM8740; Thermo Fisher). Then, fragmented mRNAs (400 ng) were incubated with anti-ac^4^C (Cat. #ab252215; Abcam) for 1 h at room temperature. Afterward, the mixtures were immunoprecipitated by incubation with prewashed Protein A Magnetic Beads (Thermo Fisher) for 5 h at 4 C. In this way, magnetic bead-antibody-RNA complexes were formed. Then, the complexes were digested by proteinase K digestion buffer (Cat. #4333,793; Thermo Fisher) at 55 °C for 1 h so that the RNA combined with the antibody was eluted from the complex. Following this, the RNAs were purified, and the enrichment of PD-L1 mRNA was assessed using RT-qPCR.

### *RIP assay*

The RIP assay was utilized to investigate the interaction between PD-L1 and NAT10 in DLD-1 and SW480 cells using the Imprint RIP Kit (Merck Millipore, Billerica, MA, USA). In brief, cells were lysed in RIP buffer for 30 min at 4 °C. Then, the cell supernatant was incubated with magnetic beads (Merck Millipore) bound with IgG and NAT10 (Cat. #ab194297; Abcam) antibodies for 4 h at 4 °C. Following the incubation, the beads underwent washing and elution. Proteinase K was then introduced to eliminate proteins at 55 °C for 30 min. The RNA was extracted, and qPCR was conducted to assess PD-L1 expression.

### *Dual-luciferase reporter assay*

Wild-type sequences of PD-L1 were amplified and cloned into pGL3 vectors (Promega, Madison, WI, USA). Their Mutant (MUT) sequences were synthesized and cloned into pGL3 vectors. DLD-1 and SW480 cells were co-transfected with the WT or MUT plasmids together with empty or NAT10 silenced vectors using Lipofectamine 3000. Luciferase activity was measured after 48 h using the dual-luciferase reporter assay system (Promega).

### *Animal study*

A total of 24 male Wistar rats (6‒8 weeks-old) were purchased from Charles River (Beijing, China) and housed in cages with 24, a 12 h alternating light/dark cycle and free access to water and food. After one-week adaptive feeding, the rats were randomly divided into four groups (*n* = 6 per group): control, kynurenine, kynurenine+lentivirus (Lv)-shNC, and kynurenine+Lv-shNAT10 groups. Lentivirus containing shNAT10 and shNC (0.2 mL, 1 × 10^9^ pfu/mL) were injected into the caudal vein of rats, respectively. Tumor volume was measured using a vernier caliper every week and quantified using the formula: Volume (mm^3^) = (length × width^2^)/2. After the fourth measurement of tumor volume, all rats were anesthetized by intraperitoneal injection with sodium pentobarbital (60 mg/kg). After deep anesthesia, the tumor was excised after cervical dislocation, and the rats were sacrificed.

### *Immunohistochemistry (IHC) assay*

Tumor tissue paraffin sections (4 μm) were incubated with anti-Ki67 (Cat. #ab15580; Abcam) and anti-PD-L1 (Cat. #ab205921; Abcam) at 4 °C overnight followed by incubating with the secondary antibody (Cat. #ab150077; Abcam) at room temperature for 0.5 h. Then, the sections were stained with diaminobenzidine solution for 3 min at room temperature. After washing using moving water and sealing, the images were visualized under a microscope.

### *Statistical analysis*

The SPSS 21.0 software was used to analyze data. Data are expressed as mean ± Standard Deviation (SD). Student's *t*-test was used for comparison between the two groups. One-way analysis of variance (ANOVA) was used for comparison among groups. Statistical analyses were performed using GraphPad Prism software (v8.0.1, GraphPad Software Inc., San Diego, CA, USA); *p* < 0.05 indicates that the difference is statistically significant.

## Results

### *Kynurenine suppressed T-cell activation and promoted immune escape*

**Fig. 1:**
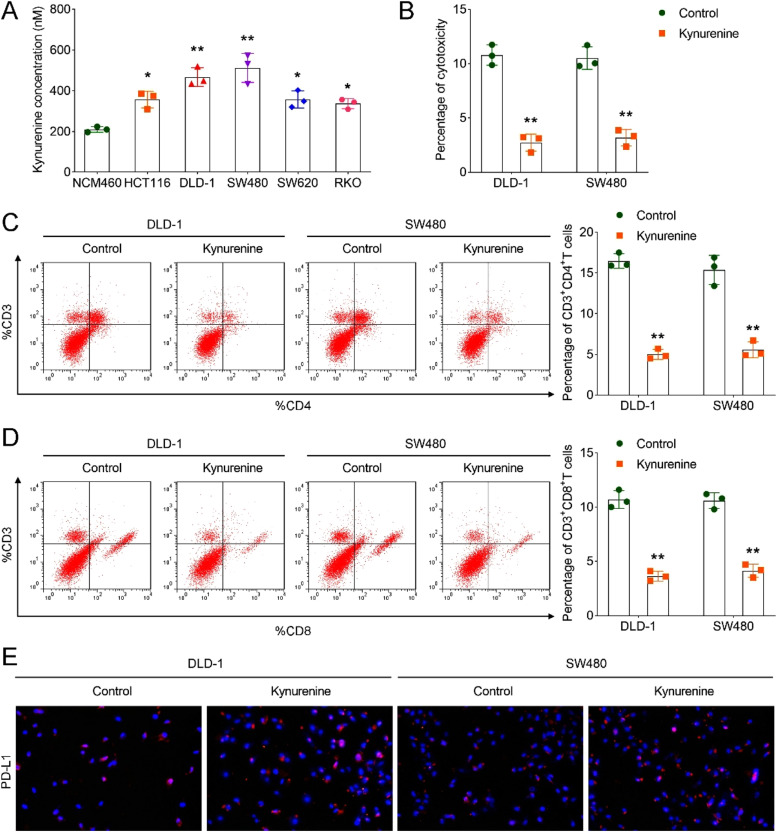
Kynurenine suppressed T-cell activation and promoted immune escape. (A) ELISA was performed to evaluate the concentration of kynurenine in CRC cells; (B) Percentage of cytotoxicity in DLD-1 and SW480 cells was assessed using MTT assay; Percentage of C, CD3+CD4+ and D, CD3+CD8+ *T*-cells in DLD-1 and SW480 cells were detected by flow cytometry; E, IF analysis of PD-L1 expression in control and kynurenine groups in DLD-1 and SW480 cells. * *p* < 0.05; ** *p* < 0.01. ELISA, Enzyme-Linked Immunosorbent Assay; MTT, 3-(4,5-dimethylthiazol-2-yl)-2,5-diphenyltetrazolium bromide; IF, Immunofluorescence; PD-L1, Programmed Death-Ligand 1.

Previous studies have highlighted the regulatory function of kynurenine in different cancers.^13^, ^14^, ^15^ In our research, the authors aimed to analyze the role of kynurenine in CRC and the underlying mechanism. ELISA results revealed that CRC cells (HCT116, DLD-1, SW480, SW620, and RKO) exhibited elevated kynurenine concentration in comparison to the NCM460 cells (Fig. 1A). DLD-1 and SW480 cells were chosen for further analysis. MTT assay was performed to assess the cell cytotoxicity in DLD-1 and SW480 cells. The findings suggested that the kynurenine treatment decreased the percentage of cytotoxicity in DLD-1 and SW480 cells (Fig. 1B), suggesting that kynurenine treatment was beneficial to the survival of DLD-1 and SW480 cells. CD3 T-cells are regulatory T-cells and CD8 T-cells are cytotoxic T-cells, both of which could reflect immune level.^16^ In further analysis, results showed that the kynurenine group downregulated the percentage of CD3^+^CD4^+^ and CD3^+^CD8^+^ *T*-cells (Fig. 1C and D), indicating that kynurenine treatment suppressed T-cell activation. PD-L1 is one of the most critical checkpoint pathways for tumor-induced immune suppression.^17^ IF results implied that kynurenine administration increased PD-L1 expression in DLD-1 and SW480 cells (Fig. 1E), suggesting that kynurenine promoted immune escape in DLD-1 and SW480 cells.

### *Kynurenine promoted NAT10-mediated ac^4^C mRNA modification*

**Fig. 2:**
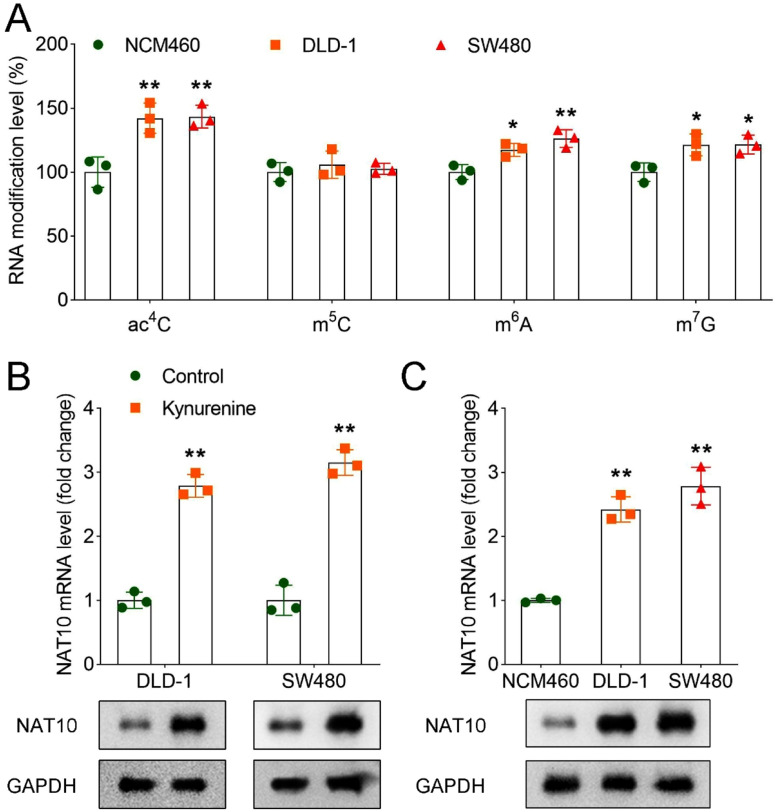
Kynurenine promoted NAT10-mediated ac^4^C mRNA modification. (A) RNA modification levels of ac^4^C, m^5^C, m^6^A, and m^7^G in NCM460, DLD-1, and SW480 cells; (B) The mRNA and protein levels of NAT10 in the control and kynurenine groups in DLD-1 and SW480 cells were assessed using the RT-qPCR and Western blot assays; (C) RT-qPCR and Western blot were performed to detect the mRNA and protein expression of NAT10 in NCM460, DLD-1, and SW480 cells. * *p* < 0.05; ** *p* < 0.01. RT-qPCR, Reverse Transcription-Polymerase Chain Reaction; NAT10, N-Acetyltransferase 10; ac^4^C, N4-acetylcytidine; m^5^C, 5-methylcytosine; m^6^A, N6-methyladenosine; m^7^G, 7-methylguanosine.

Previous studies have confirmed the existence of multiple RNA modifications in CRC.^8^^,^^18^^,^^19^ In this study, the authors wanted to explore which RNA modifications promoted PD-L1-mediated immune escape, so we performed a dot blot assay to analyze different RNA modification levels. Results indicated that ac^4^C, m^6^A, and m^7^G levels were upregulated in DLD-1 and SW480 cells compared with NCM460 cells (Fig. 2A), whereas m^5^C level showed no differences among different cells. Because the differences in ac^4^C modification were most pronounced in NCM460 and CRC cells, the authors chose ac^4^C modification for subsequent studies. NAT10 is the main ac^4^C “writer”. Further outcomes demonstrated that the kynurenine group increased NAT10 mRNA and protein levels in DLD-1 and SW480 cells in comparison to the control group (Fig. 2B). Besides, compared with the NCM460 cells, DLD-1 and SW480 cells showed increased NAT10 mRNA and protein levels (Fig. 2C).

### *NAT10 inhibition improved T-cell activation and suppressed immune escape*

**Fig. 3:**
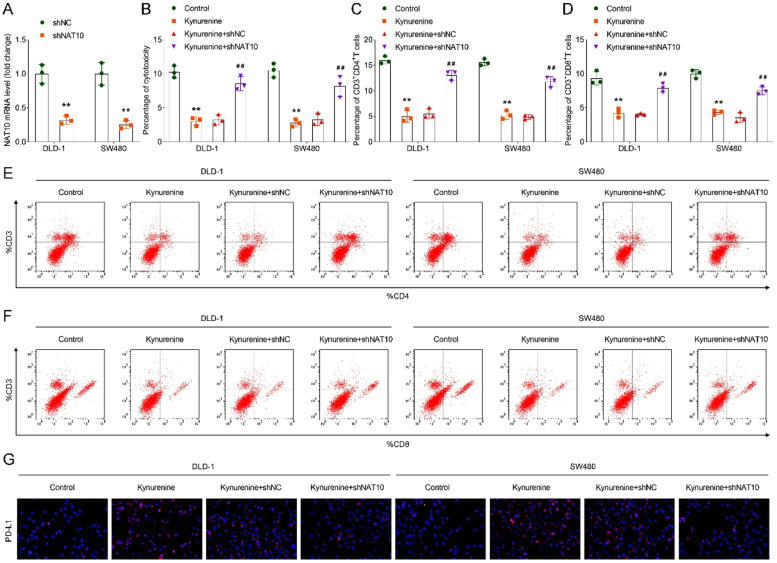
NAT10 inhibition improved T-cell activation and suppressed immune escape. (A) The mRNA level of NAT10 in DLD-1 and SW480 cells after NAT10 inhibition was assessed via RT-qPCR; (B) Percentage of cytotoxicity in DLD-1 and SW480 cells in each group was assessed by MTT assay; Percentage of C, CD3+CD4+ and D, CD3+CD8+ *T*-cells in DLD-1 and SW480 cells; Flow cytometry was performed to analyze the percentage of E, CD3+CD4+ and F, CD3+CD8+ *T*-cells in each group; G, IF analysis of PD-L1 expression in each group in DLD-1 and SW480 cells. ** *p* < 0.01 vs. the control group; ## *p* < 0.01 vs. the kynurenine+shNC group. NAT10, N-acetyltransferase 10; MTT, 3-(4,5-dimethylthiazol-2-yl)-2,5-diphenyltetrazolium bromide; IF, Immunofluorescence; PD-L1, Programmed Death-Ligand 1.

After transfecting shNAT10 into DLD-1 and SW480 cells, the mRNA expression of NAT10 was suppressed (Fig. 3A). In addition, compared with the shNC group, loss of NAT10 increased the percentage of cytotoxicity, CD3^+^CD4^+^, and CD3^+^CD8^+^ *T*-cells in DLD-1 and SW480 cells (Fig. 3B‒F), suggesting that NAT10 deficiency improved T-cell activation. Moreover, NAT10 inhibition suppressed the expression of PD-L1 (Fig. 3G) in DLD-1 and SW480 cells, indicating that immune escape was inhibited.

### *NAT10 bound with the mRNA of PD-L1 in DLD-1 and SW480 cells*

**Fig. 4:**
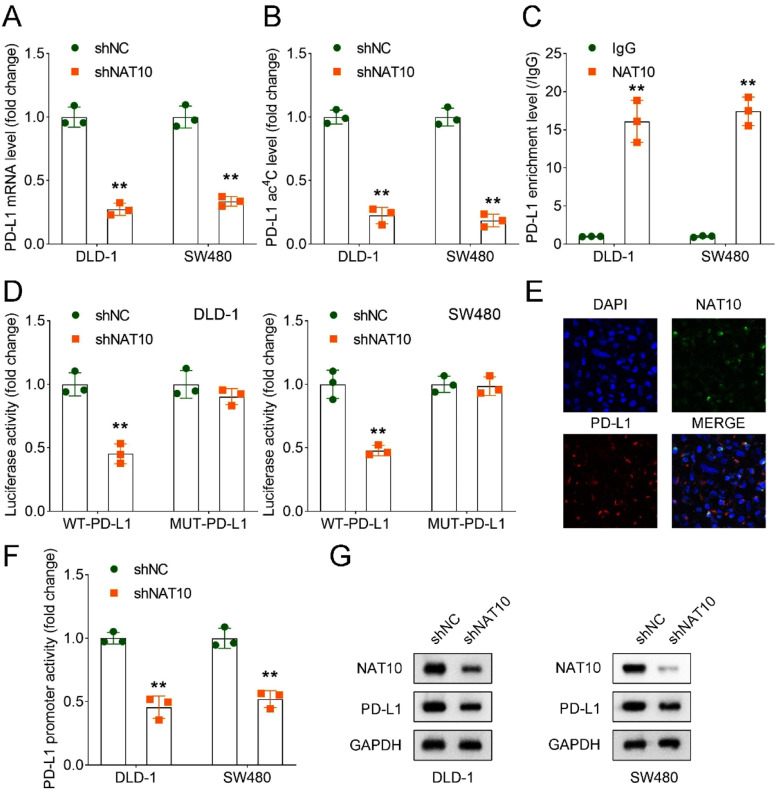
NAT10 bound with the mRNA of PD-L1 in DLD-1 and SW480 cells. (A) RT-qPCR was used to detect the expression of PD-L1 in shNC and shNAT10 groups; (B) ac^4^C-RIP assay was conducted to reveal the PD-L1 ac^4^C level in sh-NC and sh-NAT10 groups in DLD-1 and SW480 cells; (C) RIP assay was conducted to examine the interaction between NAT10 and PD-L1 in DLD-1 and SW480 cells; (D) Dual-luciferase reporter assay was performed to evaluate the binding of NAT10 and PD-L1 in DLD-1 and SW480 cells; (E) The protein distribution of PD-L1 and NAT10 in DLD-1 and SW480 cell lines was analyzed by IF assay; (F) PD-L1 promoter activity was measured by a dual-luciferase reporter assay; (G) Western blot analysis of NAT10 and PD-L1 protein levels in shNC and shNAT10 groups in DLD-1 and SW480 cells. ** *p* < 0.01. RT-qPCR, Reverse Transcription-Polymerase Chain Reaction; NAT10, N-acetyltransferase 10; shRNA, short hairpin RNA; ac^4^C, N4-acetylcytidine; RIP, RNA immunoprecipitation; IF, Immunofluorescence; PD-L1, Programmed Death-Ligand 1.

In the subsequent mechanism study, the authors wanted to explore whether NAT10 affected immune escape by affecting the ac^4^C level expression of PD-L1. After silencing of NAT10, the PD-L1 mRNA and ac^4^C levels were inhibited (Fig. 4A and B). RIP assay suggested that NAT10 bound with the mRNA of PD-L1 in DLD-1 and SW480 cells (Fig. 4C). Dual-luciferase reporter assay showed that NAT10 was specifically bound to PD-L1 in DLD-1 and SW480 cells (Fig. 4D). IF staining suggested that NAT10 co-located with PD-L1 in DLD-1 and SW480 cells (Fig. 4E). In addition, NAT10 inhibition suppressed PD-L1 promotor activity in DLD-1 and SW480 cells (Fig. 4F), suggesting that NAT10 promoted the transcription of PD-L1. Moreover, the loss of NAT10 inhibited the protein levels of NAT10 and PD-L1 in DLD-1 and SW480 cells (Fig. 4G).

### αPD-L1 treatment reversed the suppressed T-cell activation and the promoted immune escape induced by NAT10 overexpression

**Fig. 5:**
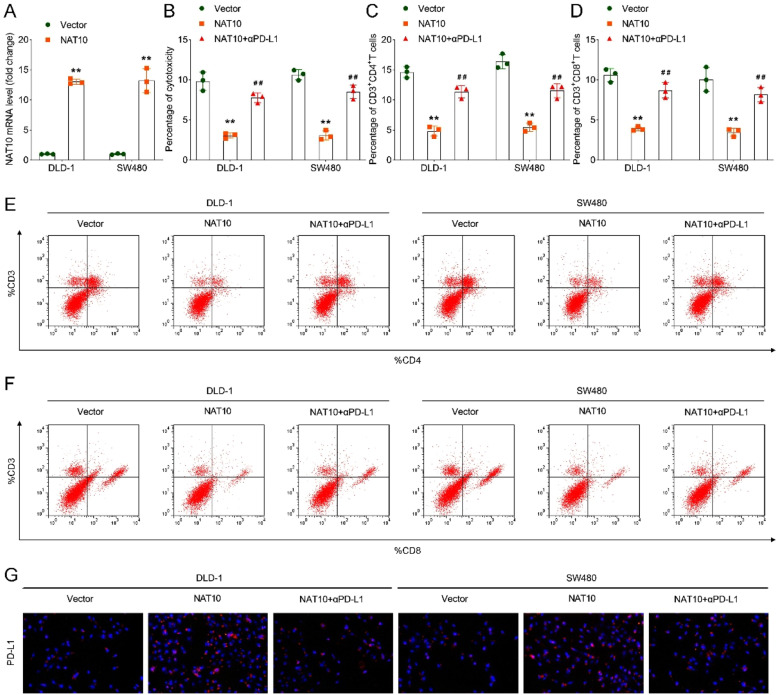
αPD-L1 treatment reversed the suppressed T-cell activation and the promoted immune escape induced by NAT10 overexpression. (A) The mRNA level of NAT10 in DLD-1 and SW480 cells after NAT10 overexpression was assessed by RT-qPCR; (B) Percentage of cytotoxicity in DLD-1 and SW480 cells in each group was assessed by MTT assay; Percentage of C, CD3+CD4+ and D, CD3+CD8+ *T*-cells in each group in DLD-1 and SW480 cells; Flow cytometry was performed to analyze the percentage of E, CD3+CD4+ and F, CD3+CD8+ *T*-cells in each group; G, IF analysis of PD-L1 expression in each group in DLD-1 and SW480 cells. ** *p* < 0.01 vs. the vector group; ## *p* < 0.01 vs. the NAT10 group. NAT10, N-acetyltransferase 10; MTT, 3-(4,5-dimethylthiazol-2-yl)-2,5-diphenyltetrazolium bromide; IF, Immunofluorescence; PD-L1, Programmed Death-Ligand 1.

In further rescue studies, the authors transfected NAT10 overexpression and the empty vectors into DLD-1 and SW480 cells, and the results showed that the expression of NAT10 was upregulated in NAT10 overexpression group compared with the vector group (Fig. 5A). Additionally, in comparison to the vector group, overexpression of NAT10 decreased the percentage of cytotoxicity and the percentage of CD3^+^CD4^+^ and CD3^+^CD8^+^ *T*-cells in DLD-1 and SW480 cells (Fig. 5B‒F). Besides, NAT10 overexpression increased the expression of PD-L1 in DLD-1 and SW480 cells (Fig. 5G). However, after treatment with the PD-L1 inhibitor, αPD-L1, the above results were all reversed.

### *NAT10 deficiency reversed the promoted tumor growth induced by kynurenine*

**Fig. 6:**
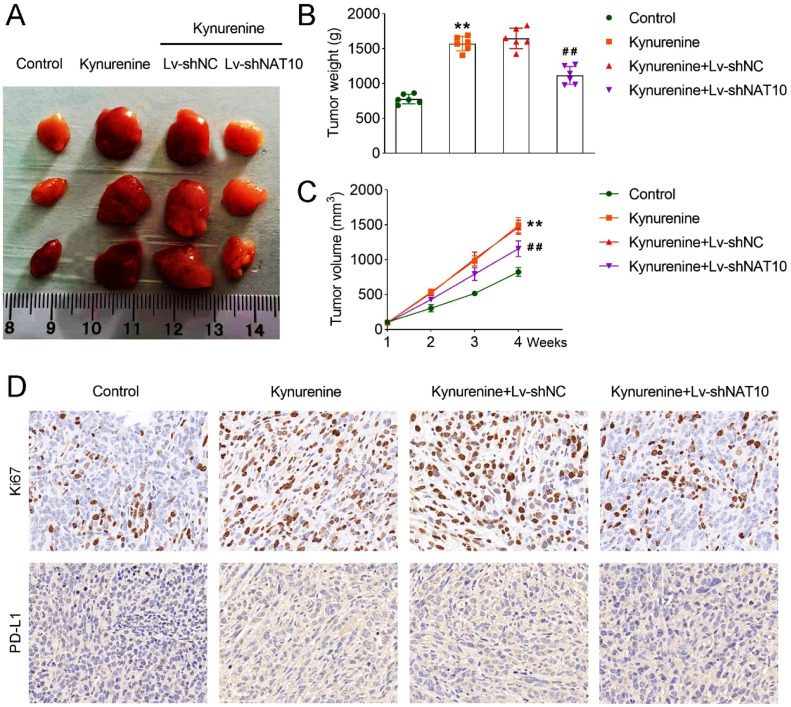
NAT10 deficiency reversed the promoted tumor growth induced by kynurenine. (A) The represent images of tumors from rat of each group; (B) Tumor weight of each group; (C) Tumor volume was measured weekly; (D) IHC was used to assess Ki67 and PD-L1 protein level in tumors. ** *p* < 0.01 vs. the control group; ## *p* < 0.01 vs. the kynurenine+Lv-shNC group. NAT10, N-acetyltransferase 10; IHC, Immunohistochemical; PD-L1, Programmed Death-Ligand 1.

In subsequent trials, the authors established the tumor-bearing rat model to explore the role of kynurenine and NAT10 in vivo. Results indicated that kynurenine treatment increased the tumor size, weight, and volume, and these results were reversed after silencing of NAT10 (Fig. 6A‒C). IHC analysis indicated that the kynurenine group showed upregulated Ki67 and PD-L1 protein levels, and the results were restored after NAT10 inhibition (Fig. 6D).

## Discussion

Kynurenine is the main metabolite of tryptophan in mammals, serving as a direct precursor to kynurenic acid, anthranilic acid, and 3-hydroxykynurenine. In this study, the authors found that the concentration of kynurenine in CRC cells was increased. Consistent with these results, previous studies indicate that kynurenine levels in tissues and plasma from CRC patients are upregulated.^13^^,^^20^ Similarly, increasing evidence finds that kynurenine is linked to other digestive tract cancers, such as esophagus, gastric, and pancreatic cancers.^14^^,^^21^^,^^22^ Besides, dysregulation of kynurenine has previously been reported implicated in breast cancer.^23^ In addition, Lee et al.^24^ demonstrate that the kynurenine-tryptophan ratio is a promising biomarker for bladder cancer surveillance.

PD-L1 is encoded by the Programmed Cell Death Protein 1 (PDCDL1) gene and is found on chromosome 9 in humans at position p24.1.2.^25^ In certain cells, minimal expression levels of PD-L1 play a role in maintaining tissue homeostasis during pro-inflammatory responses.^26^ Conversely, PD-L1 may be overexpressed due to the influence of oncogenic driver events.^27^ Thus, the blockade of PD-1/PD-L1 in antitumor immunotherapy shows huge potential in cancer treatment. In the current study, kynurenine administration decreased cytotoxicity and immune function; however, immune escape was promoted after kynurenine treatment in CRC cells, manifested by increased PD-L1 expression. The kynurenine pathway is suggested to be a critical mechanism utilized by tumor cells in evading immune surveillance to facilitate their proliferation and metastasis.^15^ A previous study indicates that the inhibition of the expression level and the enzyme activity of IDO, a tryptophan catabolic enzyme, effectively suppresses the development of chemically induced pretumor lesions in the colon.^28^ Besides, l-kynurenine downregulates T-lymphocyte hypoxia signaling to participate in cancer immune evasion.^5^ Zhang et al.^29^ discover that kynurenine inhibits T-cell infiltration and promotes immune escape by regulating the expression of Siglec-15 in head and neck squamous cell carcinoma. Hornigold et al.^30^ indicate that in renal cancer, widespread kynurenine pathway dysregulation occurs, leading to tumor immune evasion.

Research on ac^4^C modification in CRC is limited. In the present study, the authors found that NAT10-mediated ac^4^C modification was increased in CRC cells, which was consistent with a previous study.^8^ Zhang et al., demonstrate that NAT10 mediated mRNA acetylation modification patterns are associated with colon cancer progression and microsatellite status.^31^ Interestingly, research implies that microRNA (miR)-6716–5p promotes CRC cell migration and invasion by inhibiting the expression of NAT10.^32^ Moreover, NAT10-mediated ac^4^C modification facilitates the progression of other kinds of cancer, such as gastric cancer, bladder cancer, and cervical cancer.^33^, ^34^, ^35^ Besides, the authors also found that NAT10 inhibition improved T-cell activation and suppressed immune escape induced by kynurenine, suggesting that kynurenine suppressed immune function and facilitated immune escape via promoting the expression of NAT10. The authors for the first time discovered the effect of kynurenine on NAT10-mediated ac^4^C modification in CRC. Similarly, IDO1 and TDO2 catalyze kynurenine production, thus promoting the progression of CRC via compromising host immunosurveillance.^36^ In further analysis, the authors found that NAT10 is bound with the mRNA of PD-L1 in CRC cells. Besides, NAT10 promoted the transcription of PD-L1. Similar to these results, NAT10 leads to enhanced transcription and increased expression of PD-L1 by promoting the acetylation of nucleophosmin 1 in different cancer cells, including human breast cancer, melanoma, and colorectal cancer cells.^37^ Besides, targeting tryptophan catabolism in ovarian cancer could attenuate PD-L1 expression.^38^ In tumor-bearing rats, kynurenine treatment promoted tumor growth, while NAT10 deficiency reversed these results. These results were consistent with the in vitro results and suggested that kynurenine promoted tumor growth through facilitating the expression of NAT10. Similarly, previous studies in prostate and bladder cancer discover that NAT10-mediated ac^4^C modification promotes the tumor growth of xenografted mice.^33^^,^^39^

In conclusion, kynurenine promoted the immune escape of CRC cells via NAT10-mediated ac^4^C acetylation of PD-L1, which might provide new insights into the treatment of in CRC. However, there are several limitations in this study. Firstly, the authors only used DLD-1 and SW480 cells for analysis, and it is necessary to investigate more CRC cells in future studies. Besides, the underlying mechanism of kynurenine on CRC may be complex, including but not limited to NAT10-mediated ac^4^C modification. Moreover, the authors lack data from clinical studies. The authors will further address these deficiencies as experimental conditions permit in the future.

## Ethics approval and consent to participate

This study was approved by the Ethics Committee of The Affiliated Bozhou Hospital of Anhui Medical University (No. 2024233). All animal experiments should comply with the ARRIVE guidelines. All methods were carried out in accordance with relevant guidelines and regulations.

## Consent for publication

Not applicable.

## Availability of data and materials

The datasets used and/or analyzed during the current study are available from the corresponding author upon reasonable request.

## Authors' contributions

All authors participated in the design, interpretation of the studies and analysis of the data, and review of the manuscript. ZBW drafted the work and revised it critically for important intellectual content; MMY, RHZ, ML and JP were responsible for the acquisition, analysis and interpretation of data for the work; ZGW made substantial contributions to the conception or design of the work. All authors read and approved the final manuscript.

## Funding

The work was supported by the Anhui Provincial University Natural Science Research Project in 2023 under grant number 2023AH053303; Bozhou Municipal Science and Technology Bureau 2024 Key R&D Project under grant number bzzc2024025.

## Conflicts of interest

The authors declare no conflict of interest.
